# Establishment and operation of a Good Manufacturing Practice-compliant allogeneic Epstein-Barr virus (EBV)-specific cytotoxic cell bank for the treatment of EBV-associated lymphoproliferative disease

**DOI:** 10.1111/bjh.13051

**Published:** 2014-07-26

**Authors:** Mark A Vickers, Gwen M Wilkie, Nicolas Robinson, Nadja Rivera, Tanzina Haque, Dorothy H Crawford, Jacqueline Barry, Neil Fraser, David M Turner, Victoria Robertson, Phil Dyer, Peter Flanagan, Helen R Newlands, John Campbell, Marc L Turner

**Affiliations:** 1 Scottish National Blood Transfusion ServiceAberdeen, London, UK; 2 University of AberdeenAberdeen, London, UK; 3 Royal Free HospitalLondon, UK; 4 University of EdinburghEdinburgh, UK; 5 Scottish National Blood Transfusion ServiceEdinburgh, UK; 6 New Zealand Blood ServiceAuckland, New Zealand

**Keywords:** cell therapy, Epstein-Barr virus, cytotoxicity, lymphoproliferative disease

## Abstract

Epstein-Barr virus (EBV) is associated with several malignancies, including post-transplant lymphoproliferative disorder (PTLD). Conventional treatments for PTLD are often successful, but risk organ rejection and cause significant side effects. EBV-specific cytotoxic T lymphocytes (CTLs) generated *in vitro* from peripheral blood lymphocytes provide an alternative treatment modality with few side effects, but autologous CTLs are difficult to use in clinical practice. Here we report the establishment and operation of a bank of EBV-specific CTLs derived from 25 blood donors with human leucocyte antigen (HLA) types found at high frequency in European populations. Since licensure, there have been enquiries about 37 patients, who shared a median of three class I and two class II HLA types with these donors. Cells have been infused into ten patients with lymphoproliferative disease, eight of whom achieved complete remission. Neither patient with refractory disease was matched for HLA class II. Both cases of EBV-associated non-haematopoietic sarcoma receiving cells failed to achieve complete remission. Thirteen patients died before any cells could be issued, emphasizing that the bank should be contacted before patients become pre-terminal. Thus, this third party donor-derived EBV-specific CTL cell bank can supply most patients with appropriately matched cells and most recipients have good outcomes.

Epstein-Barr virus (EBV) infects most humans, sometimes causing infectious mononucleosis (Visser *et al*, 2014), but always establishing a life-long latent infection in B lymphocytes. This state is usually considered to be asymptomatic, but has been associated with both predisposition to autoimmune diseases (Almohmeed *et al*, 2013; Hanlon *et al*, 2014) and several malignancies (Chen *et al*, 2012; Roschewski & Wilson, 2012), including post-transplant lymphoproliferative disorder (PTLD). Initial treatment for PTLD comprises reduction of immunosuppression and rituximab (Messahel *et al*, 2006), with or without chemotherapy (Heslop, 2009). However, the condition continues to have a high mortality from both refractory disease and complications of treatment. In addition, as it often occurs in the context of otherwise successful transplants, it may cause donated organs to be wasted.

Data from infected but healthy individuals show the importance of cytotoxic T cell (CTL) responses in the control of latent EBV infection (Hislop *et al*, 2007). The risk of PTLD correlates inversely with anti-EBV T cell activity in patients, particularly in children who have not previously encountered the virus (Abu-Elmagd *et al*, 2009). Cellular therapy was first shown to be effective against PTLD in the form of unmanipulated donor lymphocyte infusions after bone marrow transplantation (Papadopoulos *et al*, 1994). Autologous or donor-derived CTLs directed specifically against EBV were subsequently developed and are a non-toxic but still effective form of treatment (Heslop *et al*, 2010; Bollard *et al*, 2012). However, several factors have worked against their widespread adoption as a therapeutic modality. In particular, the low incidence of PTLD (1–5%) means that the generation of prophylactic autologous CTLs in advance for patients is not usually cost-effective, although the refinement of risk factors may make prediction worthwhile (Uhlin *et al*, 2014). The lengthy period, typically 2–3 months, necessary for CTL culture makes their generation post-diagnosis difficult in a clinically useful timeframe. Finally, the perceived efficacy of chemotherapeutic regimens containing rituximab has rendered autologous anti-EBV CTLs to be considered unviable as a clinical product. Nevertheless, about one-third of patients with a diagnosis of PTLD continue to die either from PTLD itself or complications of treatment (Burns *et al*, 2013; Caillard *et al*, 2013; Styczynski *et al*, 2013; Uhlin *et al*, 2014).

Thus, several strategies are being employed to enhance the effectiveness of CTLs (Bollard *et al*, 2014). In haematopoietic stem cell transplantation (HSCT), donor lymphocytes responding to EBV can be collected by apheresis, selected by cytokine secretion and infused within days of diagnosis into patients (Moosmann *et al*, 2010; Uhlin *et al*, 2012; Gerdemann *et al*, 2013). An alternative approach that we, and others, have used is to pre-prepare a bank of EBV-specific CTLs from third party donors. This approach was described in 2001 (Haque *et al*, 2001) and developed over subsequent years, (Haque *et al*, 2002; Wilkie *et al*, 2004), with a trial reporting overall responses of 64% at 5 weeks and 52% (14 complete and three partial in 33 patients) at 6 months (Haque *et al*, 2007, 2010). Other groups have reported the use of third party donors with similar outcomes in PTLD and we have summarized these data in Table I (Sun *et al*, 2002; Barker *et al*, 2010; Doubrovina *et al*, 2012; Leen *et al*, 2013). However, cell banks developed in the context of research laboratories had to be abandoned for clinical use in Europe after 2005, as they were not made in accordance with Good Manufacturing Practice (GMP) and relevant European Commission (EC) legislation, most recently Regulation No 1394/2007 ‘designed to ensure the free movement of advanced therapy products within Europe, to facilitate access to the EU market and to foster the competitiveness of European companies in the field, while guaranteeing the highest level of health protection for patients’. In response, the Wellcome Trust and Scottish National Blood Transfusion Service (SNBTS) funded the set up of a GMP-compliant cell bank, which was licenced by the Medicines and Healthcare products Regulatory Agency (MHRA) to start supplying CTLs in February 2012. Here we report the operation and outcomes of the bank after 2 years of operation.

**Table 1:** Previous experience of third party derived CTLs in the treatment of PTLD.

	*n*	Partial response	Complete response	Overall response rate (%)
Sun *et al* (2002)	2	0	2	100
Haque *et al* (2007, 2010)	33	3	14	52
Doubrovina *et al* (2012)	19	0	13	68
Leen *et al* (2013)	9	4	2	67
Totals	63	7	31 (47%)	60

## Establishment of cell bank

Peripheral blood mononuclear cell (PBMC) donations were kindly provided by donors located in New Zealand in order to minimize the risk of Creutzfeldt–Jacob disease transmission. The New Zealand Blood Service provided access to a human leucocyte antigen (HLA)-typed, pre-screened apheresis donor panel. Each donor gave informed consent and was required to test positive for antibodies against EBV viral capsid antigen, preferably be blood group O and test negative for antibodies to Hepatitis B virus (HBV), Hepatitis C virus (HCV), human immunodeficiency virus (HIV), human T-lymphotropic virus type I (HTLV1) and syphilis. In addition, donors were selected to maximize the probability of HLA class I and II matches and minimize the number of mismatches. This was performed by taking the HLA types of 304 patients from the East of Scotland renal transplant waiting list and performing simulations using 200 donors from the Auckland, New Zealand platelet apheresis donor panel. Antigens shared between putative donor-recipient pairs were scored as ‘2’ for two antigens shared at any one locus, ‘1’ for one antigen shared and ‘0’ if no antigens were shared. At a minimum low-resolution level mismatch (HLA-A, -B, DRB1 ‘111’ or better), it was found that 15 well-selected donors could cover 57% of the patient population. This rose to 85% with 25 donors, but further increases in the size of the putative donor panel did not significantly increase the number of matches. The panel size chosen was therefore 25 and donors are listed in Table II. Due to the mismatch minimization procedure used, HLA homozygotes are over-represented.

**Table 2:** HLA types of unrelated donors.

CTL ID	HLA Class I						HLA Class II				Issues
	A^*^		B^*^		C^*^		DRB1^*^		DQB1^*^		
NZ020	23:01	68:01	15:01	18:01	03:03	07:01	11:01	15:01	03:01	06:02	
NZ026	29:02	29:02	44:03	51:07	14:02	16:01	07:01	11:01	02:02	03:01	
NZ111	02:01	02:01	13:02	27:05	02:02	06:02	01:01	15:01	05:01	06:02	
NZ153	01:01	02:01	08:01	15:01	03:04	07:01	03:01	13:01	02:01	06:03	
NZ177	02:01	02:01	51:01	57:01	02:02	06:02	13:01	15:01	06:02	06:03	
NZ200	01:01	31:01	08:01	40:01	03:04	07:01	03:01	04:04	02:01	03:02	
NZ209	01:01	02:01	08:01	44:02	05:01	07:01	03:01	11:03	02:01	03:01	2
NZ294	02:01	03:01	14:02	44:03	08:02	16:01	07:01	13:02	02:02	06:09	
NZ298	01:01	29:02	07:02	44:03	07:02	16:01	07:01	07:01	02:01	02:01	
NZ327	02:01	02:01	44:02	44:02	05:01	05:01	11:01	12:01	03:01	03:01	
NZ332	02:01	24:02	40:01	44:02	03:04	07:04	11:01	13:02	03:01	06:04	
NZ417	32:01	68:01	44:02	44:02	05:01	07:04	13:01	14:01	05:03	06:03	
NZ449	02:01	02:01	07:02	44:02	05:01	07:02	04:01	15:01	03:02	06:02	
NZ578	01:01	03:01	08:01	57:01	06:02	07:01	03:01	07:01	02:01	03:03	
NZ610	03:01	68:02	14:02	51:01	08:02	15:02	01:01	13:03	03:01	05:01	
NZ612	01:01	02:01	18:01	35:02	04:01	07:01	11:04	11:04	03:01	06:03	
NZ666	01:01	24:02	08:01	15:18	03:03	07:01	03:01	11:06	02:01	03:01	
NZ675	26:01	26:01	49:01	51:01	07:01	16:02	01:01	13:02	05:04	06:04	
NZ771	11:01	26:01	27:05	35:01	02:02	04:01	04:01	11:01	03:01	03:01	
NZ806	01:01	01:01	08:01	08:01	07:01	07:01	03:01	03:01	02:01	02:01	2
NZ823	02:01	32:01	15:01	15:01	03:03	03:03	03:01	15:01	02:01	06:02	
NZ873	01:01	03:01	07:02	08:01	07:01	07:02	03:01	15:01	02:01	06:02	5
NZ898	02:01	32:01	14:01	44:02	05:01	08:02	04:01	07:01	02:02	03:02	
NZ932	02:01	26:08	40:02	51:01	02:02	15:02	01:01	11:01	03:01	05:01	1
NZ988	02:01	03:01	44:02	44:02	07:04	08:02	04:01	04:01	03:01	03:02	1

HLA, human leucocyte antigen; CTL ID, cytotoxic T lymphocytes identification code.

Approximately 25–50 × 10^9^ mononuclear cells (MNCs) were collected from each of 25 donors into 10 bags by apheresis, cryopreserved in 10% dimethyl sulfoxide (DMSO), then shipped to the UK in vapour phase liquid nitrogen by an accredited shipper with temperature logging throughout. On receipt, cells were transferred to vapour storage. Culture conditions have been described previously (Wilkie *et al*, 2004), but briefly: an aliquot of MNCs was infected with purified EBV, manufactured by a US Food and Drug Administration (FDA) registered facility at the Baylor College of Medicine (Houston, TX, USA), and lymphoblastoid cell lines (LCLs) established over 4–8 weeks. LCLs express the type III latency pattern proteins associated with PTLD and are also efficient antigen-presenting cells, expressing both HLA class I and II as well as several co-stimulatory ligands. LCLs were irradiated and used for antigen stimulation on a revived aliquot of MNCs at an initial ratio of 40:1 MNC:LCL. Subsequent weekly stimulations used a 4:1 CTL:LCL ratio with 20 iu/ml interleukin- 2 (IL2) added thrice weekly from 14 d. EBV-specific CTLs were expanded over approximately 2 months, then cryopreserved in 10% DMSO frozen in Origen CryoStore bags of 50 × 10^6^ or 150 × 10^6^ cells using a controlled rate freezer, before placing in nitrogen vapour-phase storage at <−150°C. Final product samples passed bacterial, mycoplasma, viral and endotoxin testing as negative or not detected. Bacterial testing was by SNBTS standard procedure using the BacT/Alert 3D system (BioMérieux, Basingstoke, UK). Mycoplasma 28-d cultural testing was according to test requirements of PhEur-2008, by Mycoplasma Experience (Reigate, UK). Viral nucleic acid amplification testing was of HCV, HIV and HBV by SNBTS. Endotoxin levels were tested using Biowhittaker LAL methodology (Cambrex Bio Science Walkersville, Inc., Walkersville, MD, USA) by SNBTS. Samples were immunophenotyped for CD3, CD4, CD8, CD19 and CD56 and were required to be <2% CD19^+^. Cytotoxic function and anti-viral specificity were tested using a flow cytometric-based assay based on previously reported methods (Derby *et al*, 2001). CTLs were incubated at 20:1, 10:1, 5:1 and 2·5:1 effector:target ratios, in 96 round-welled plates, in RPMI medium supplemented with 20% fetal bovine serum (FBS), for 4 h with PKH67-stained targets. Target cells, at 10^4^/well, comprised autologous LCLs, EBV-uninfected phytohaemoagglutinin (PHA)-stimulated lymphoblasts, HLA-mismatched LCLs and K562 (a natural killer cell target). After a further 20-min incubation with 7-aminoactinomycin D (7AAD) to stain non-viable cells, samples were run through a flow cytometer. The percentage of double positive stained cells in test wells minus the respective target-only wells, gave an indication of killing after incubation. CTLs were required to demonstrate functionality, by having autologous LCL killing >10%, and specificity, by <10% or lower killing of the other target cells. The documentation in each batch record was reviewed by the quality department prior to authorization for release.

The MHRA certified compliance with GMP and awarded a ‘Specials’ Licence, allowing the release of an Advanced Therapy Medicinal Product (ATMP) in October 2011. A final quality sign-off was performed in February 2012. At licensure only three CTL lines were available for use; this number gradually increased over the next 18 months to the full complement of 25. After an enquiry is made to the bank, the referring clinicians are requested to complete an Initial Patient Enquiry form, which includes information on HLA types and any anti-HLA antibodies. If a suitable HLA match is found, a blood sample is requested to verify the HLA type, detect any anti-HLA antibodies and also check that no non-specific cell killing is seen. A sample of proposed CTLs is tested in a repeat cytotoxicity assay against autologous LCLs to confirm cytotoxic function (positive control) and patient phytohaemagglutinin (PHA)–stimulated blasts to check that they are not killed. Cells are then shipped to a Human Tissue Authority (HTA) licenced receiving centre, along with information sheets and infusion instructions. CTLs are infused at doses of 1−2 × 10^6^/kg body weight as a course of four infusions given at weekly intervals. All forms are downloadable from our website (http://www.scotblood.co.uk/about-us/clinical-services/cytotoxic-t-cell-therapy.aspx). Correspondence is generally carried out from nss.ctlbank@nhs.net. A non-profit making, cost recovery charge of £12 500 is charged for the supply of cells.

## Operation of the cell bank

### Enquiries before licensure

In the year before the certification of compliance with GMP, eleven enquiries about CTLs were received, which had to be declined as we had not yet received a Manufacturer's licence. Nine of the eleven patients had confirmed EBV+ PTLD and six of these patients are believed to have died. Of the three who survived, one patient travelled to America for CTL therapy and two responded to conventional therapies. The two other enquiries comprised a presumptive diagnosis of chronic active EBV infection and a girl with a congenital immunodeficiency with previous EBV-associated lymphoproliferative disorder being prepared for an unrelated donor peripheral progenitor cell transplant. The former diagnosis was changed to angioimmunoblastic T cell lymphoma and secondary activation of EBV, with a fatal outcome. We were asked to grow CTLs from a prospective donor for the second, but the donation centre was unable to supply PBMC. The transplant went ahead with prophylactic rituximab used against the risk of PTLD, but the patient died of thrombotic thrombocytopenic purpura and adenoviral infection. Thus, overall, eight of the eleven enquiries pre-licensure had a fatal outcome.

### Enquiries resulting in issue of CTLs

**Figure 1:**
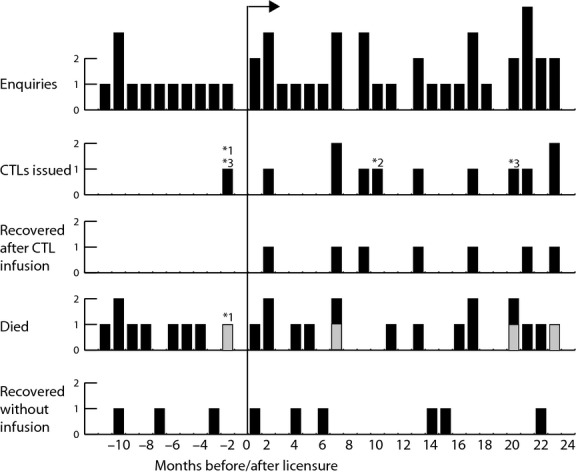
Timeline of bank summarizing enquiries and outcomes. Months before licensure are shown as negative numbers on the horizontal axis, and months after as positive numbers. Cases are indicated by stacked bars and are ordered by time at which initial contact was made with the bank. *1 indicates a directed donation from the father of the patient; the cytotoxic T lymphocytes (CTLs) were infused after licensure. *2 indicates that the issued CTLs have not yet been infused. *3 indicates that the patients had EBV-associated non-haematopoietic sarcoma, the other recipients had post-transplant proliferative disorder.

In the 21 months since licensure, and 3 months post manufacturing certification, but pre-licensure, 37 enquiries were received. The frequency of enquiries increased slightly during this time (Fig1). Twelve of these enquiries resulted in the issue of CTLs (Table III). Nine of the twelve (Patients 1, 3, 4, 7, 8, 9, 10, 11, 12) were cases of PTLD. Of these, one was CD20- and rituximab-resistant (Patient 1), one had concomitant graft-versus-host disease (GVHD) precluding reduction of immunosuppression (Patient 3), one was unfit for chemotherapy because of recent necrotizing fasciitis and multi-organ failure (Patient 4) and six cases had the CTLs used to consolidate reduction of immunosuppression and chemotherapy. Four patients had central nervous system (CNS) disease, where rituximab penetration is known to be poor. Of these nine, seven went into complete remission, while two (Patients 3, 9) had progressive disease and died within weeks of the infusions. Neither of these two had an HLA class II match (see below), although both had three HLA class I matches. Both also had relatively rapidly progressive disease at the time of infusions. In a further two cases (Patients 2, 5), the CTLs were planned to be used as a bridge to allogeneic HSCT for EBV+ lymphoproliferation associated with congenital immunosuppression, although only one of these has been infused at the time of writing.

**Table III:** Enquiries resulting in issue of cells.

Patient	Age (years)	Sex	Reason for immunosuppression	Disease: site	HLA match/mismatch (HLA-A,B,C,DR,DQ)	Outcome
1	8	Female	Mismatched cord blood transplant for aplastic anaemia	PTLD: nasopharynx, lung, liver, CNS	1,1,1,1,1 0,0,0,0,0	Complete response
2	1	Female	Congenital immune deficiency pre-cord blood transplant	PTLD: CNS, lung, kidney, liver, lymph node	2,2,2,1,2 0,0,0,1,0	Complete response
3	14	Male	VUD PBSCT for chronic granulomatous disease	PTLD: lymph node	2,0,1,0,0 0,1,1,1,0	Progressive disease, died
4	53	Male	Renal transplant	PTLD: CNS	1,1,1,1,1 0,0,0,0,0	Complete response
5	1	Female	Di George syndrome with GVHD from maternal cell graft	High EBV titres pre-transplant	1,0,0,1,1 1,2,2,1,1	Not yet transplanted, CTLs not yet infused
6	5	Male	Cardiac transplant	Leiomyosarcoma: lung	Paternal donor	Partial response, fatal infection
7	55	Female	VUD PBSCT for CML	PTLD: CNS	1,1,1,1,1 1,1,0,1,1	Complete response
8	24	Female	Cardiac transplant	PTLD: lymph node	1,1,1,1,1 1,1,0,1,1	Complete response
9	4	Male	VUD PBSCT for congenital immune deficiency	PTLD: pulmonary, liver	1,0,2,0,01,2,0,2,2	Progressive disease – died
10	1	Female	Hepatic transplant	PTLD: stomach, duodenum	1,1,1,1,1 1,1,1,1,1	Complete response
11	13	Female	Cardiac, renal transplants	PTLD: lymph node. EBV+ spindle cell: CNS	2,1,1,2,2 0,1,1,0,0	Complete response for PTLD, but spindle cell tumour progressed.
12	56	Male	VUD PBSCT for aplastic anaemia	PTLD: lymph node	1,0,1,1,1 0,2,1,1,1	Complete response

Match/mismatch data are given at antigen level.

PTLD, post-transplant proliferative disorder; CNS, central nervous system; VUD, volunteer unrelated donor; PBSCT, peripheral blood stem cell transplant; GVHD, graft-versus-host disease; CML, chronic myeloid leukaemia; EBV, Epstein-Barr virus; HLA, human leucocyte antigen; CTLs, cytotoxic T lymphocytes.

Cytotoxic T lymphocytes were also infused into two cases of EBV-associated non-haematopoietic sarcomas. One (Patient 6) had chemotherapy-resistant EBV-associated leiomyosarcoma post-cardiac transplant and the CTLs were derived from the father of the patient. In the computerized tomography scan taken 6 weeks after the last infusion, several of the lesions had decreased in size and there were no new lesions, which was felt to represent a partial response. However, the patient died of an infection shortly thereafter, not thought to be related directly to the CTL infusions. The other (Patient 11) had a CNS spindle cell tumour. The case was originally discussed with the bank and it was decided not to infuse cells in view of the lack of an evidence base for the use of CTLs in this condition. The patient subsequently developed typical PTLD. CTLs were infused with the PTLD responding well, but the spindle cell tumour progressed and the patient died.

### Enquiries terminated by deaths of patients

Thirteen patients died before any cells could be issued. It took an average of 6 d (range 2–11) between completion of the Initial Patient Request Form and death. In ten cases, the patients died before cells could be shipped. In three cases, the patient or their families decided during the selection process that palliative care was preferable to active treatment and matched CTLs were not shipped.

### Enquiries resulting in a clinical decision not to issue CTLs

In 11 patients, the treating clinicians decided not to pursue CTL therapy after the initial enquiry. Six did not have PTLD. In three of these six cases it was decided there was no malignancy present: in one of these cases infectious mononucleosis was diagnosed. In two cases, there was an EBV-driven malignant disorder (EBV+ diffuse large B cell lymphoma of the elderly, haemophagocytic lymphohistiocytosis), but it was decided by the referring clinicians not to proceed with infusions. The other six cases of PTLD for whom infusions were not pursued improved with conventional therapy after contacting the cell bank.

### HLA matching of patients with bank donors

After receipt of the Initial Patient Enquiry form, the HLA types of CTLs and patients at HLA-A, -B, -C, -DRB1 and -DQB1 were reviewed. The maximum number of shared antigens was therefore 10. No locus was preferentially matched. Where a patient had an antibody specific to a CTL antigen, this CTL was not considered further in the allocation process. Where possible, allelic level matching between CTLs and patients was assessed to optimize CTL activity. Blood samples were requested to monitor patients after each of the four weekly infusions and 12 weeks afterward. No CTL-directed HLA-specific antibodies have been detected post-infusion in any of the patients.

**Figure 2:**
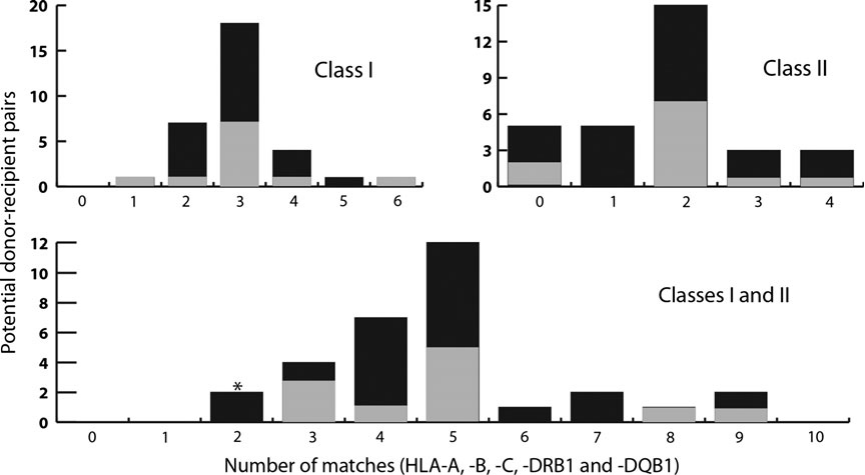
Human leucocyte antigen matching of donors with recipients. The number of matches at five human leucocyte antigen (HLA) loci are shown as histograms of the best matched prospective recipient–donor pairs. Matches that resulted in cytotoxic T lymphocyte infusions are shown in grey, those that did not in black. For two pairs, information was only available at HLA class I.

To date, 36 HLA matching requests have been processed and 32 allocation reviews completed. The minimum number of antigens shared for both HLA class I and class II combined was 2/10 and the maximum 9/10 (Fig2). For the CTLs infused, the minimum number of antigens shared was 3/10 and the maximum 9/10. Figure2 understates the degree of matching somewhat, as information was only available at class I loci for two of the pairs (neither patient received infusions). There was a median of three class I matches and two class II matches. The degree of matching for class II improved with time as the numbers within the bank built up, but this effect was not apparent for class I. Those that were issued had a slightly higher degree of matching (3·0 vs. 2·8 class I and 1·9 vs. 1·8 class II).

Results of matching for CTLs infused are given as number of HLA-A, B, C, DR and DQB1 matches respectively (Table III), so that, for example 1,0,2,1,0 indicates a single matched allele/antigen at HLA-A and DR, two alleles/antigens matched at HLA-C and none matched at HLA-B and DQB1. For issue, we recommended at least one HLA class I and one HLA class II match, based on our previous findings that response rates correlated with number of matches and CD4 cell counts, although it was the final decision of the treating clinicians whether to proceed to infusion. Only 4/36 matches failed these criteria and all failed on having no HLA class II match. Two of these (2,0,1,0,0 and 1,0,2,0,0) were issued. It seems plausible that this poor HLA class II matching contributed to these two patients (Patients 3, 9) being the only ones with PTLD to show no clinical response.

### Side effects

For ten of the 11 recipients, no side effects were reported. One case (Patient 3) suffered from possible GVHD. Five hours after the first infusion of anti-EBV CTLs, two areas of petechiae were noted on the front and back of trunk. The following day, the rash spread to *c*. 50% of the body area. Although dissimilar to previous skin GVHD and not biopsied, the most likely diagnosis was felt to be GVHD. Treatment with topical steroid and tacrolimus resolved the rash within 24 h. Subsequent infusions were not associated with rashes, although the patient died shortly thereafter of refractory PTLD.

## Discussion

We describe the establishment and operation of a GMP-compliant, third-party, EBV-specific CTL bank manufactured under all relevant EC regulation. By careful choice of the HLA types of donors, a bank of only 25 donors was able to supply cells to patients, with no request turned down solely on the basis of poor HLA matching. In the first 2 years of its operation, CTLs were issued to 12 patients and infused into 11. The outcomes of patients receiving cells for lymphoproliferative disease have been relatively good, with 8/10 achieving complete remission and only two dying of refractory PTLD. However, neither patient receiving cells for EBV-associated non-haematopoietic sarcoma responded well.

The main advantage of third party-derived cells compared to autologous cells is relatively rapid availability after a diagnosis has been made. We set up the bank to issue cells within 14 d after initial contact. It is therefore disappointing that 13 patients died within 11 d of contact. Most of the patients about whom we were contacted were failing conventional treatments at the time of request. Several potentially avoidable delays were noted: in addition to the process of cytotoxicity testing, it often took over a week to obtain authorization for funding. In retrospect, it might have been better if the bank had been contacted earlier in the courses of these diseases. However, the median time to death was 16 d in a recent series of 40 patients with PTLD, with around half of patients dying of their disease (Uhlin *et al*, 2014).

The favourable outcomes of the patients receiving cells for PTLD and the high proportion of patients dying pre-licensure support the view that CTLs comprise an effective treatment modality. Third party donor-derived CTLs might be expected to be less efficacious than fully HLA-matched CTLs, but the outcomes from this bank, as well as other centres (Doubrovina *et al*, 2012), have reported broadly similar outcomes to the use of autologous cells. However, the patients who died almost certainly had an intrinsically worse prognosis than those who received cells. It is also notable that six patients with PTLD did not proceed to receiving infusions, yet their disease improved in response to conventional treatments. Overall, it seems likely that CTLs contributed to the good outcomes of recipients. Nevertheless, the series we report is descriptive and does not represent a formal test of efficacy.

Of the four patients who received cells but died, there are two notable features. First, two had a non-haematopoietic sarcoma-like histology. Much less is known about the expression pattern of EBV genes in these tumours, which is likely to differ from PTLD. Furthermore, the specificities of CTLs directed against the LCLs used as antigenic stimulation include non-EBV encoded, lymphoid-specific antigens (Long *et al*, 2009), probably lacking in sarcomas. In addition, these sarcomas are able to down-regulate expression of major histocompatibility complex molecules (Berghuis *et al*, 2009). These theoretical difficulties are borne out by our poor results and alternative approaches for these patients are needed. The two patients with PTLD who were refractory to CTL infusions were notable for the lack of any HLA class II matches and relatively aggressive clinical presentations. We previously reported poor results from CTLs with low proportions of CD4 cells (Haque *et al*, 2007), so these cases support the view that CD4 cells are important for anti-PTLD efficacy. It is, however, unclear whether the effects of CD4 cells are due to directly mediated cytotoxicity or cytokine-mediated help provided to CD8 cells. Doubrovina *et al* (2012) also reported that clinically aggressive disease responded less well to CTL infusions.

If the efficacy of EBV-specific CTLs is accepted, the question arises as to which patients should be chosen for their use. At present, two classes of patients seem particularly appropriate and the recipients of our cells exemplify these indications. First, PTLD resistant to conventional chemotherapy, often CNS disease or CD20- and, consequently, resistant to rituximab. The second class is situations where immunosuppression cannot be withdrawn, either because it is constitutive or where its reduction threatens graft, infection or GVHD.

Indeed, given the lack of side effects compared to conventional chemotherapeutic agents (Cruz *et al*, 2010) and their apparent efficacy, third party anti-EBV CTLs may also be indicated in other situations. Although rituximab can be effective for PTLD, the short-term response rate is only about 60–80% (Messahel *et al*, 2006; Burns *et al*, 2013; Caillard *et al*, 2013; Styczynski *et al*, 2013) and the longer-term prognosis may be worse (Heslop *et al*, 2010). Use of CTLs might therefore be considered at an earlier stage in the therapeutic pathway. EBV is increasingly recognized as causing lymphomas in situations other than post-transplant, including secondary to other causes of immunosuppression, such as HIV, drugs, such as methotrexate, or non-immunosuppressed individuals (Dojcinov *et al*, 2011; Ok *et al*, 2013). The efficacy of CTLs remains to be tested in these contexts.

This report adds to the evidence that anti-EBV CTLs have few side effects. However, one case of likely skin GVHD was observed in the 2 d after one of the infusions. While we cannot be sure that the CTLs were the cause of the rash, it is notable that mild, transient skin GVHD has been reported from other centres (Prockop *et al*, 2012; Leen *et al*, 2013). No cases affecting other sites have been reported, although GVHD of similarly mild severity affecting other organs may well not be observable. It remains possible that a more severe case of GVHD might occur in the future.

Overall, we report that a third party anti-EBV CTL bank can be operated in accordance with GMP under current EU legislation. Outcomes from patients receiving CTLs have been good, especially if both HLA class I and II loci were matched. However, a high proportion of patients died in the period after the bank was alerted, but before any cells could be issued. In the future, this CTL bank should be useful for a wider range of patients within Europe.
